# Forgotten Radicals in Biology

**Published:** 2008-12

**Authors:** Rochette Luc, Catherine Vergely

**Affiliations:** *Laboratoire De Physiopathologie Et Pharmacologie Cardiovasculaires Experimentales, Facultes De Medecine Et Pharmacie, Dijon Cedex, France*

**Keywords:** free radicals, reactive oxygen nitrogen species, cardiovascular, physiopathology, hydrogen disulphide

## Abstract

Redox reactions play key roles in intra- and inter-cellular signaling, and in adaptative processes of tissues towards stress. Among the major free radicals with essential functions in cells are reactive oxygen species (ROS) including superoxide anion (O_2_^•-^), hydroxyl radical (^•^OH) and reactive nitrogen species (RNS) such as nitric oxide (^•^NO). In this article, we review the forgotten and new radicals with potential relevance to cardiovascular pathophysiology. Approximately 0.3% of O_2_^•-^ present in cytosol exists in its protonated form: hydroperoxyl radical (HO_2_^•^). Water (H_2_O) can be split into two free radicals: ^•^OH and hydrogen radical (H^•^). Several free radicals, including thiyl radicals (RS^•^) and nitrogen dioxide (NO_2_^•^) are known to isomerize double bonds. In the omega-6 series of poly-unsaturated fatty acids (PUFAs), cis-trans isomerization of γ-linolenate and arachidonate catalyzed by RS^•^ has been investigated. Evidence is emerging that hydrogen disulphide (H_2_S) is a signaling molecule *in vivo* which can be a source of free radicals. The Cu-Zn superoxide dismutase (SOD) enzyme can oxidize the ionized form of H_2_S to hydro-sulphide radical: HS^•^. Recent studies suggest that H_2_S plays an important function in cardiovascular functions. Carbonate radical, which can be formed when ^•^OH reacts with carbonate or bicarbonate ions, is also involved in the activity of Cu-Zn-SOD. Recently, it has been reported that carbonate anion were potentially relevant oxidants of nucleic acids in physiological environments. In conclusion, there is solid evidence supporting the formation of many free radicals by cells leading which may play an important role in their homeostasis.

## INTRODUCTION

Reactive oxygen species (ROS) are ubiquitous reactive derivates of O_2_ metabolism in all biological systems. They are some of the newest additions to the family of second-messenger molecules. They are traditionally regarded as injurious cellular by-products with the potential to damage all cell components. However, there is now convincing evidence that redox reactions play key roles in adaptation of tissue towards stress, and in intra- and intercellular signalling. Among the major free radicals with essential functions in cells are reactive oxygen species (ROS), including superoxide anion (O_2_^•-^), hydroxyl radical (^•^OH), and reactive nitrogen species (RNS) such as nitric oxide (^•^NO) (1). In addition, reactive species that were practically forgotten in biology up to the recent years have recently been brought into the fore. In this article, we highlight a review of the forgotten and new radicals with potential relevance in pathophysiology.

## HYDROPEROXYL RADICAL: HO_2_^•^

**Figure 1:**
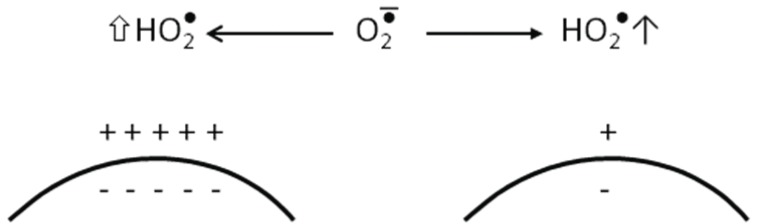
Importance of proton gradient acidity of the outer surface of the inner membrane of the mitochondria on the protonation of superoxide. Cardiolipin with its negative charges (in inner membrane → Acidification of the surface) (5).

HO_2_^•^ is usually termed either hydroperoxyl radical or perhydroxyl radical. It is the protonated form of superoxide. Approximately 0.3% of superoxide present in the cell cytosol exists in the protonated form (2). A number of groups, studying on mitochondrial biology, have reported that the efficiency of the respiratory chain enzymes can be altered with aging due to at least two factors: oxidative damage to its constituent proteins and change in their environment. The major microenvironmental change with age may be reduced levels of cardiolipin in the inner membrane, and complex IV is particularly dependant on cardiolipin as a cofactor (3). Cardiolipin is the only negatively charged phospholipid present in significant quantity in the inner membrane (4). Lower cardiolipin levels result in a less acid aqueous phase at the membrane surface and hence in a reduction of the transformation of O_2_^•-^ to HO_2_^•^. Restoration by dietary supplementation of cardiolipin in old rats with acetyl-L-carnitine is associated with an increase in the rate of oxidative stress (5) (Figure 1).

Since HO_2_^•^ is present in all the compartments where O_2_^•-^ is produced, its functions should be analyzed. Recently, it has been reported that molecular hydrogen can selectively reduce ROS *in vitro* (6). To identify the free radicals species that H_2_ reduces, Ohsawa *et al* (6) studied the effects of H_2_ on electron spin resonance signals of spin-trapping reagents (5,5-dimethyl-1-pyrroline-N-oxide: DMPO). Only ^•^OH-derived signals were decreased by H_2_ treatment. It is likely that H_2_ has a number of advantages as a potential antioxidant; it can penetrate biomembranes and diffuse into the cytosol and cellular organites. At the pH7.4 the ratio of [O_2_^•-^]/[HO_2_^•^] is 100/1.

## HYDROGEN RADICAL: H^•^, THIYL RADICALS: RS^•^, HYDROSULPHIDE RADICAL: HS^•^

**Figure 2:**
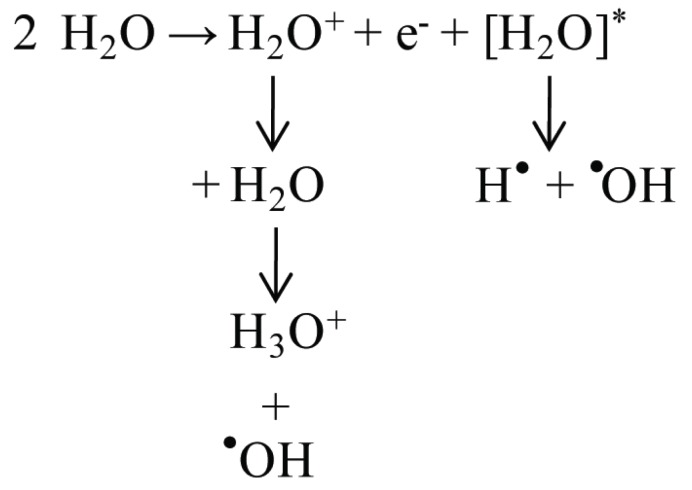
Production of oxygen free radicals during radiolysis.

The simplest atom is the element hydrogen; containing one proton (atomic number one, masse number one) and one electron. During radiolysis of aqueous solutions, energy is absorbed by water to produce ionization and excitation (Figure 2).

It has been suggested that H_2_ protects cells against strong oxidative stress by scavenging ^•^OH but it is possible that H_2_ directly or indirectly reduces other oxidant species. It is important to remember that water H_2_O can be split into two free radicals: hydroxyl radical ^•^OH and hydrogen radical H^•^. Reactions of ^•^OH are: hydrogen abstraction, addition and electron transfer. One example of H^•^ abstraction is the reaction of ^•^OH with alcohols. The ^•^OH abstracts H^•^ and combines with it to form water. In biological systems, the major role of hydrogen is in determining the pH of intracellular and extracellular compartments. An acid may be defined as a donor of hydrogen ions. It is widely accepted that the transmembrane difference in the electrochemical potential of hydrogen ions is a major intermediate in the cellular energy transduction. This potential is generated by redox driven proton pumps. Recently, the pH hypothesis has been evoked in myocardial protection. Cohen *et al* (7) reported that postconditioning prevents mitochondrial permeability transition pore (MPTP) formation by maintaining acidosis during the first minutes of reperfusion as reoxygenated myocardium produce ROS that activate protective signaling to inhibit MPTP formation after pH normalization. One obvious limitation of the present study was the absence of pH measurements.

Both, intracellular and extracellular redox states of thiols play an important function in antioxidant protection. Biological action of cellular thiol compounds is related to the activity of hydrosulfide group which determines their ability to participate in detoxicant and antioxidant reactions. In antioxidant reactions, thiols undergo one-electron oxidation with the formation of thiyl radicals (RS^•^).

RSH → RS^•^ + H^+^ + e^-^

Thiyls radicals may be generated in reactions with other oxygen free radicals and with H_2_O_2_.

$\mathrm{RSH}+O_{2}^{\bar{•}}+H^{+}\to {\mathrm{RS}}^{•}+H_{2}O_{2}$

2 RSH + H_2_O_2_ → 2 RS^•^ + 2 H_2_O

RSH + ^•^OH → RS^•^ + H_2_O

In the presence of trace amounts of transition metal ions, thiol compounds are oxidized to form thiyl radicals and reactive oxygen species.

RSH + Me^n^ → RS^•^ + Me^n-1^ + H^+^

Thiol compounds can exert pro-oxidative action by the reduction of transition metal such as ion: Fe^+3^ to Fe^2+^ leading to the formation of thiyl radicals and generation of superoxide radical anion.

RSH + Fe^3+^ → RS^•^ + Fe^2+^ + H^+^

${\mathrm{Fe}}^{2+}+O_{2}\to {\mathrm{Fe}}^{3+}+O_{2}^{\bar{•}}$

Another important characteristic of thiyl radicals is their ability to abstract hydrogen atoms from the molecules such as

1. from lipids, initiating peroxidative lipid damage
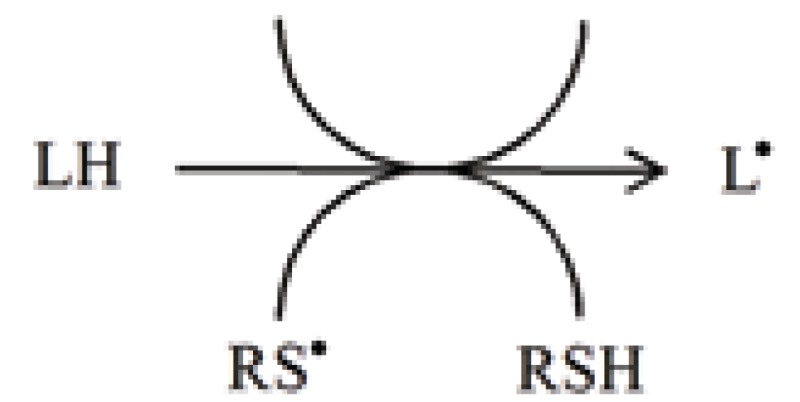
2. from organic compounds
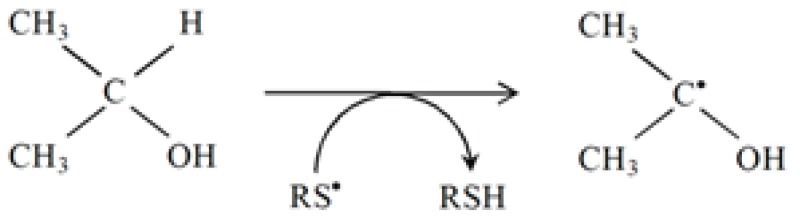
3. from vitamin: Ascorbate
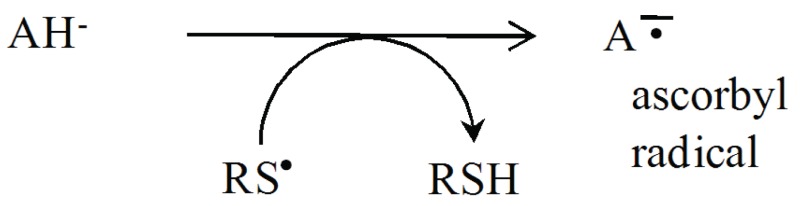

The rate and efficiency of the removal of RS^•^ has a critical effect on the actions of thiols in the cellular compartments.

Several free radicals, including thiyl radicals and nitrogen dioxide (NO_2_^•^) are known to isomerizes double bonds. The cis-trans isomerization by RS^•^ is an efficient process. In the omega-6 series of poly-unsaturated fatty acids (PUFAs), cis-trans isomerization of γ-linolenate and arachidonate catalyzed by RS^•^ has been investigated (8).

In this context, crucial questions remain to be answered by free radical research involving lipids in a biological environment defining the site of action of these radicals and their characteristics. Human and animals are continuously exposed in their environment to numerous exogenous thiol compounds and related disulfides; thiol compounds being present in food, pollutants and products during biodegradation of sulfur -containing substances.

A novel concept of "gasotransmitter" has been recently proposed. Gasotransmitters are small molecules composed of endogenous gases. Then production and metabolism are enzymatically regulated. Following the identification of ^•^NO and CO as gasotransmitters, H_2_S has been suggested as the third gasotransmitter. H_2_S is released from the desulfuration of L-cysteine via two pyridoxal-5’-phosphate (PLP) - dependent enzymes in mammalian tissues.

Evidence is emerging that hydrogen sulfide (H_2_S), essentially hydrogen thiol H-SH is a signaling molecule *in vivo*. Thiols can generate free radicals. The Cu-Zn SOD enzymes can oxidize the ionized form of H_2_S to hydro-sulfide radical: HS^•^. Recent evidence suggests that H_2_S plays an important function in cardiovascular and brain functions, acting as an intracellular messenger (9). At physiologically relevant concentrations H_2_S relaxes vascular tissues, an effect mediated by the activation of ATP sensitive K^+^ (K_ATP_) (10). Furthermore, the endogenous production of H_2_S in the cardiovascular system seems be regulated by ^•^NO. On human aorta smooth muscle cells, recent findings support the hypothesis that endogenously produced H_2_S plays a fundamental role in cell proliferation and survival (11). Recently, it has been reported (12) that the delivery of H_2_S at the time of reperfusion limits infarct size and preserves left ventricle function in an *in vivo* model of myocardial ischemia-reperfusion. This protective effect is associated with an inhibition of myocardial inflammation and a preservation of mitochondrial structure. According to the biochemistry and physical properties of H_2_S in presence of H_2_O, it remains possible that H_2_S protects cells and tissue against oxidative stress by directly or indirectly reducing oxidant species in living cells. For instance H_2_S may induce cytoprotection via H_2_ or hydro-sulfide radical HS^•^. It is anticipated that future studies will reveal the molecular mechanisms underlying the radical mechanism implicated in the intracellular effects of H_2_S.

## NITROGEN DIOXIDE: ^•^NO_2_

NO_2_^•^ is a radical having the unpaired electron located about 53% at the nitrogen atom and to 23.5% at each of the two oxygen atoms. Two ^•^NO_2_ radicals can be dimerized to create the high reactive compound: dinitrogen tetraoxide.

Nitrogen dioxide is a major atmospheric pollutant. However, recently it has been reported that nitrogen dioxide can be generated *in vivo* by a variety of sources such as system myeloperoxidases and its participation has been evoked in atherosclerosis or amyotrophic lateral sclerosis in relation with the metabolism of vitamin C.

Under physiological conditions, dues to the slow steady-state levels of ^•^NO and O_2_, the antioxidation of ^•^NO to ^•^NO_2_ is slow in aqueous area such as plasma or cytosol.

2^•^NO + O_2_ → 2^•^NO_2_

^•^NO is nine times more soluble in a hydrophobic solvent than in water, enabling the reaction ^•^NO with O_2_ to exist. This reaction is about 300 times more rapid within membranes than in the surrounding aqueous medium (13). In these conditions, the autoxidation of ^•^NO can occur. Moreover, in acidic conditions, a specific reaction is possible; the nitrite/HOCl couple is potential source of endogenous ^•^NO_2_. This radical acts on the antioxidative mechanisms. It has been reported that exposure of human plasma to low amounts of NO_2_ was associated to a decrease in antioxidants (ascorbate, α-tocopherol) (14). If these antioxidants are depleted, peroxidation of lipids occurs and might contribute to the toxicity of ^•^NO_2_.

In biological systems where multiple free radicals were present, the recombination reaction of ^•^NO_2_ with another radical is very fast.

NO2•+O2•¯→O2NOO−

Its conjugative acid: peroxynitric acid is a strong oxidant so it is a highly toxic compound. Ascorbic acid is a very important compound that rapidly scavenges ^•^NO_2_.

Terminating the chain reaction sequence, glutathione (GSH) is also an important intracellular ^•^NO_2_ antagonist and its scavenging activity of GSH appears particularly important in the mitochondria (15).

As we previously reported, ^•^NO_2_ is known to able to isomerize double bonds. However, consideration of kinetic data and product studies on the reaction of ^•^NO_2_ led to the conclusion that in well-oxygenated cells, catalysis of cis-trans isomerization by ^•^NO_2_ should be an unimportant process, because oxygen reacts rapidly with a carbon-centered radical intermediate, thus leading mostly to lipid peroxidation (16).

## CARBONATE RADICAL ANION: CO_3_^•-^


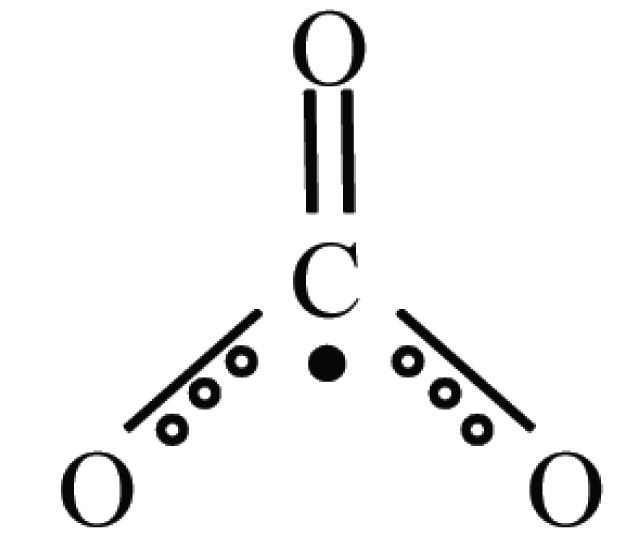


The carbonate radical anion has been produced by radiolysis of aqueous solutions of bicarbonate/carbonate (17). More recently it has been reported that the reaction between peroxynitrite and carbon dioxide may formed carbonate radical anion and nitrogen dioxide. Thus, under physiological conditions, where the concentration of CO_2_ is high, rapid reaction of peroxynitrite with CO_2_ occurs.

ONOO−+CO2→ONOOCO2−→NO2•+CO3•¯

Carbonate radicals can be also formed when ^•^OH reacts with carbonate or bicarbonate ions. Bicarbonate levels in blood plasma are high (25 mM) making reaction possible (18, 19).

CO32−+OH•→CO3•¯+OH−

A wide variety of biomolecules can be oxidized by carbonate radical. It has been reported that it can abstract ^•^H from cysteine.

$\mathrm{cys}-\mathrm{SH}+{\mathrm{CO}}_{3}^{\bar{•}}\to {\mathrm{HCO}}_{3}^{-}+{\mathrm{cysS}}^{•}$

Carbonate radical is also involved in the activity of Cu Zn SOD (20). The (bi) carbonate-dependent peroxidative activity of SOD1 has been explained on the bases of (bi) carbonate to carbonate radical anion occurs at the enzyme active site by reaction with the H_2_O_2_-induced enzyme - copper bound oxidant.

Cu II−SOD1/OH•+bi carbonate→Cu II−SOD1−OH+CO3•¯

CO_3_^•-^ has a much longer half-life than ^•^OH and can therefore diffuse further from the enzyme active site and can oxidatively modify distant cellular targets.

During myocardial reperfusion, the increased flux of free radicals (21-24), which reacts with ^•^NO enhances the formation of ONOO^-^. These peroxynitrite ions are decomposed as previously mentioned. ^•^NO_2_ and CO_3_^•-^ were formed within the heart and may exert deleterious effects. A nitroxide compound as 4-hydroxy-2,2,6,6-tetramethylpiperidine-1-oxyl (TPL) in presence of arginine significantly improved the recovery of hemodynamic function of hearts following ischemia-reperfusion (25). The kinetics of the reaction of CO_3_^•-^ with TPL has been studied and it has been demonstrated that TPL is an efficient scavenger of CO_3_^•-^.

Reactions of the carbonate radical anion with transition metal ions in metalloproteins are also predictable but they have been little studied thus far. The reaction of the carbonate and dichloride radical anions with the extracellular matrix glycosaminoglycan hyaluronan (HA) had been studied *in vitro* (26). These radicals were found to react quickly with HA inducing a scission of HA.

Recently, it has been reported that carbonate anions were potentially important oxidants of nucleic acids in physiological environments. The CO_3_^•-^ radicals oxidize guanine bases of DNA through a one-electron transfer reaction process that ultimately results in the formation of stable guanine oxidation products. Base sequence effects have been explored (27); relevantly, GSH, proteins and nucleic acids are likely to be main targets at this radical.

### Clinical perspective

There is solid evidence supporting the formation of many free radicals by cells leading to the view that they play an important role in cell homeostasis (28-31). In addition to the traditional focus reactive species, recent studies of the biochemical reactions of nitric oxide, carbonate radical, and hydrogen have identified potential reactions proposed to contribute to the pathogenesis of diseases including inflammatory processes, ischemia-reperfusion and cardiovascular disorders. A special interest surrounds experimental investigations aimed at further elucidating the cellular mechanisms underlying the effects of oxidative stress in the respiratory systems and ROS-signaling pathway. Therefore, elucidating the mechanism of oxidative stress and antioxidant systems may lead us to understand the pathogenesis of different diseases and to discovery novel therapeutic strategies.
